# COVID-19: Studying dissociative experiences in a confined sample of tunisian people

**DOI:** 10.1192/j.eurpsy.2021.738

**Published:** 2021-08-13

**Authors:** E. Baklouti, A. Larnaout, K. Souabni, R. Lansari, W. Melki

**Affiliations:** 1 Psychiatry, hospital razi, la manouba, Tunisia; 2 Psychiatry D, Razi Hospital, Manouba, Tunisia

**Keywords:** COVID-19, dissociative experiences, confinment, DES-II

## Abstract

**Introduction:**

Dissociative experiences have been studied in different circumstances.

**Objectives:**

In this study we aim to analyze dissociative phenomena under a stress factor: lockdown.

**Methods:**

We conducted a cross sectional-study, using an online survey, spread during lockdown period in Tunisia, between the 2nd and the 8th of April. It was comprised of sociodemographic, geographic, medical history, confinement status and DES-II questionnaire in its french version. Age superior than 18 was the only inclusion criteria and the no respect of lockdown was the exclusion criteria. Based on former studies on DES-II, 3 sub scores have been assessed; amnesia (measures memory loss), depersonalization (sense of unreality of the self ) and absorption (the absorption has to do with one’s traumatic experiences).

**Results:**

We recruited 167 individuals; 100 women and 67 men. The most common age class was 20 to 30 years old (60.5% of the sample). The Mean total score was 11.06 which was higher than mentioned in earlier studies. The mean score was 15,11 for absorption; 5,28 for amnesia and 6,88 for depersonalization subscale. Significant differences in scores were found based on different variables. Women had higher absorption score (p=0.011). Besides people living in COVID-19 clusters had lower total score (p=0.038). Finally, people with somatic medical history showed higher total score (p=0.013), absorption score (p=0.003) and depersonalization score (p=0.012) compared to those with none.

**Conclusions:**

During lockdown, dissociative experiences showed to be more frequent. But does this mean that a resurgence in PTSD (posttraumatic stress disorder) or ASD (acute stress disorder) in the months to come.

